# Holarctic genetic structure and range dynamics in the woolly mammoth

**DOI:** 10.1098/rspb.2013.1910

**Published:** 2013-11-07

**Authors:** Eleftheria Palkopoulou, Love Dalén, Adrian M. Lister, Sergey Vartanyan, Mikhail Sablin, Andrei Sher, Veronica Nyström Edmark, Mikael D. Brandström, Mietje Germonpré, Ian Barnes, Jessica A. Thomas

**Affiliations:** 1 Department of Bioinformatics and Genetics, Swedish Museum of Natural History, 10405 Stockholm, Sweden; 2 Department of Zoology, Stockholm University, Stockholm 10691, Sweden; 3 Department of Earth Sciences, Natural History Museum, London SW7 5BD, UK; 4 Northeast Interdisciplinary Research Institute, Far East Branch, Russian Academy of Sciences, Magadan 685000, Russia; 5 Zoological Institute of Russian Academy of Sciences, Saint-Petersburg 199034, Russia; 6 Institute of Ecology and Evolution, Russian Academy of Sciences, Moscow 119071, Russia; 7 Department of Forest Mycology and Pathology, Swedish University of Agricultural Sciences, 10691 Uppsala, Sweden; 8 Operational Direction ‘Earth and History of Life’, Royal Belgian Institute of Natural Sciences, Vautierstraat 29, 1000 Brussels, Belgium; 9 School of Biological Sciences, Royal Holloway University of London, Egham, Surrey TW20 0EX, UK

**Keywords:** ancient DNA, *Mammuthus primigenius*, extinction, refugia, climate

## Abstract

Ancient DNA analyses have provided enhanced resolution of population histories in many Pleistocene taxa. However, most studies are spatially restricted, making inference of species-level biogeographic histories difficult. Here, we analyse mitochondrial DNA (mtDNA) variation in the woolly mammoth from across its Holarctic range to reconstruct its history over the last 200 thousand years (kyr). We identify a previously undocumented major mtDNA lineage in Europe, which was replaced by another major mtDNA lineage 32–34 kyr before present (BP). Coalescent simulations provide support for demographic expansions at approximately 121 kyr BP, suggesting that the previous interglacial was an important driver for demography and intraspecific genetic divergence. Furthermore, our results suggest an expansion into Eurasia from America around 66 kyr BP, coinciding with the first exposure of the Bering Land Bridge during the Late Pleistocene. Bayesian inference indicates Late Pleistocene demographic stability until 20–15 kyr BP, when a severe population size decline occurred.

## 1. Introduction

One of the greatest strengths of an ancient DNA (aDNA) approach is that it enables the study of genetic change through time. Analyses of samples across particular geographical regions through time have revealed unexpected patterns of local population extinction and recolonization [1–6]. However, while such studies are invaluable for investigating the interaction between population dynamics and local changes in the environment, it is not always clear how different lineages evolved and where recolonizing populations originated. A more comprehensive approach, encompassing the full geographical extent of a species’ distribution, is needed to fully understand its biogeographic history [7,8]. Furthermore, a large sample size is important to provide good spatio-temporal coverage and sufficient detail for the reconstruction of evolutionary events.

The woolly mammoth (*Mammuthus primigenius*) is one of the best-studied taxa in the field of aDNA. Adapted to the cold and arid steppe–tundra, mammoths were widespread during the Late Pleistocene (*ca* 116–12 kyr BP), with a range that extended from Western Europe to the northern part of North America [9]. However, the availability of well-preserved permafrost samples in Siberia and Alaska has meant that genetic studies have predominantly focused on the region of Beringia [6,10–13]. By contrast, little is known about the population structure at the western end of mammoth distribution in Europe, although aDNA extracted from a single European specimen indicated a high level of sequence divergence from other mammoth populations [6]. Furthermore, while morphological data revealed that woolly mammoths were present in Europe from around 200 kyr BP until the end of the Pleistocene [14,15], little is known about their population dynamics within Europe, nor about the extent of gene flow between European and Asian populations.

Previous genetic studies on Beringian mammoths identified two deeply divergent monophyletic mitochondrial DNA (mtDNA) lineages, clades I and II, hypothesized to have evolved in isolation on either side of the Bering Strait [6,10]. Clade I (haplogroups C, D and E in Debruyne *et al*. [11]) had a widespread distribution during the later stages of the Late Pleistocene, but appears to have originated in North America and dispersed into Eurasia during the Middle or early Late Pleistocene [6,11]. Clade II (haplogroup A in Debruyne *et al*. [11]) had a much more limited geographical distribution in eastern Siberia and is thought to have a Siberian origin [6,10]. It appears that these two genetic lineages coexisted in northeast Siberia for thousands of years before clade II disappeared at approximately 40 kyr BP [6,10]. Clade I, however, survived well into the Holocene. The last mainland populations of clade I mammoth persisted in areas of northern Siberia until *ca* 11 kyr BP [16] but, in contrast to most megafaunal species that went extinct around the Pleistocene/Holocene transition, small populations of woolly mammoth survived to the mid-Holocene, until *ca* 6 kyr on St Paul Island [17] and 4 kyr on Wrangel Island [18].

While several hypotheses have been proposed to explain the genetic changes that took place during the Late Pleistocene, a full picture is yet to emerge regarding the origin of different genetic lineages and the timing of changes in demography and genetic variation. This study has three aims. First, to establish the woolly mammoth's Late Pleistocene genetic structure across the whole of its Holarctic distribution. Second, to examine different hypotheses regarding the mammoth's evolutionary history, including levels of genetic diversity and the timing of local population turnover events, range expansions and contractions. Third, to improve the resolution of phylogenetic and demographic analyses, since previous studies on mammoth genetics have been shown to potentially lack significant molecular signal [19]. In this study, we extract and analyse mtDNA from woolly mammoth specimens from across the Holarctic, expanding the genetic sampling both spatially and temporally. We include specimens from Europe as well as from Siberia identified as Middle Pleistocene in age. Combined with previously published mtDNA sequences, the dataset comprises more than 300 mammoth specimens, thus enabling a thorough reconstruction of the species’ population history from the Late Middle Pleistocene (LMP) up until its extinction.

## 2. Material and methods

### (a) DNA analysis and radiocarbon dating

We recovered DNA from specimens of bone, tooth and tusk (*n* = 88) collected from most of the Holarctic range of the woolly mammoth (see electronic supplementary material, table S1). MtDNA amplification was performed as in Barnes *et al*. [6], targeting a 741 bp region, including the 3′ end of the cytochrome *b* gene (CytB), two tRNA genes (tRNA-Thr and tRNA-Pro), and the first hypervariable part of the control region (CR1). Pre-PCR laboratory work was performed in dedicated aDNA laboratories at Royal Holloway, University of London and the Swedish Museum of Natural History in Stockholm, following standard protocols and procedures (for details, see the electronic supplementary material). Radiocarbon dating was performed at the Oxford Radiocarbon Accelerator Unit using accelerator mass spectrometry. Radiocarbon dates were calibrated in OxCal v. 4.1 [20] with the IntCal09 calibration curve [21] (see the electronic supplementary material, tables S1 and S2).

### (b) Phylogenetic analyses

Our ancient mtDNA sequences were aligned with homologous woolly mammoth sequences available on GenBank [6,10–13,22–25] (see the electronic supplementary material, table S2 for accession numbers) in Geneious v. 5.0.1 [26]. We used Partition Finder [27] to select the best-fit partitioning scheme and DNA substitution model (see electronic supplementary material, table S3). Bayesian phylogenies were generated using MrBayes v. 3.2.1 [28], with two African elephant (*Loxodonta cyclotis*, *Loxodonta africana*) and one Asian elephant (*Elephas maximus*) sequences as outgroups (accession nos: AY359274, NC000934 and EF588275). Four chains were run for 20 million generations and sampled every 2000. BEAST v. 1.7 [29] was used to construct dated phylogenies and to estimate the dates of three specimens that were stratigraphically dated to the LMP, using the tip-dating method. For the dating analyses, the temporal signal of the data was first assessed using the date randomization test [19]. BEAST analyses were run under three different population models, for 200 million generations and sampled every 20 000. Convergence for both phylogenetic analyses was assessed in Tracer [30]. A median-joining network was constructed using Phylonet5 v. 1.0.0 (A. Helgason 2009, unpublished). Changes in phylogeographic patterns over time were visualized in GenGIS [31].

### (c) Coalescent simulations and approximate Bayesian computation

Serial coalescent simulations were run with Bayesian Serial SimCoal [32] and analysed in an approximate Bayesian computation (ABC) framework [33] using the ABC package in R [34]. Summary statistics (see electronic supplementary material, table S4) were calculated in Arlequin v. 3.5 [35]. Using the age of first reproduction as a proxy for generation time [36], we assumed a generation time of 15 years [37]. The mean mutation rate (9.56% per site/10^6^ years), transition bias (0.98) and shape parameter of gamma distribution (0.107) estimated from BEAST were used in all simulations. One million iterations were run for each scenario. Two regression methods were employed, local linear regression [38] and the neural networks algorithm [39] with 1% acceptance ratio. The latter method is recommended when highly dimensional summary statistics are used in order to transform the number of possibly correlated variables into a smaller number of variables [40]. Additional simulations with a generation time of 20 years were run to assess the effect of generation time on the outcome of the analysis.

#### (i) Models 1A and 1B

Two scenarios were simulated to examine whether a history of population isolation in multiple refugia during the previous interglacial could explain the observation of different mtDNA clades: (i) three populations (representing the three mtDNA clades) that split from each other with constant effective population size (*N*_ef_) and a uniform prior for the split time versus (ii) three populations that split from each other (as above) and experienced a simultaneous bottleneck, followed by exponential growth. *N*_ef_, split time and start time of expansions were sampled from uniform priors (for details, see the electronic supplementary material, figure S1).

#### (ii) Models 2A and 2B

We estimated the split time between the North American and Eurasian populations carrying mtDNA clade I, which could represent the time when North American individuals crossed over the Bering Land Bridge, leading to the introduction and expansion of clade I woolly mammoths in Eurasia [6,11]. In addition to the hypothesized dispersal time, subsequent gene flow in the opposite direction was assessed. Two alternative models were thus evaluated with two populations (representing North America and Eurasia) that diverged from each other: (i) without gene flow after the divergence event or (ii) with subsequent gene flow from Eurasia to North America. Uniform priors were used for *N*_ef_, split time and migration rate (for details, see the electronic supplementary material, figure S2).

## 3. Results

### (a) Mitochondrial DNA diversity

**Figure 1.**
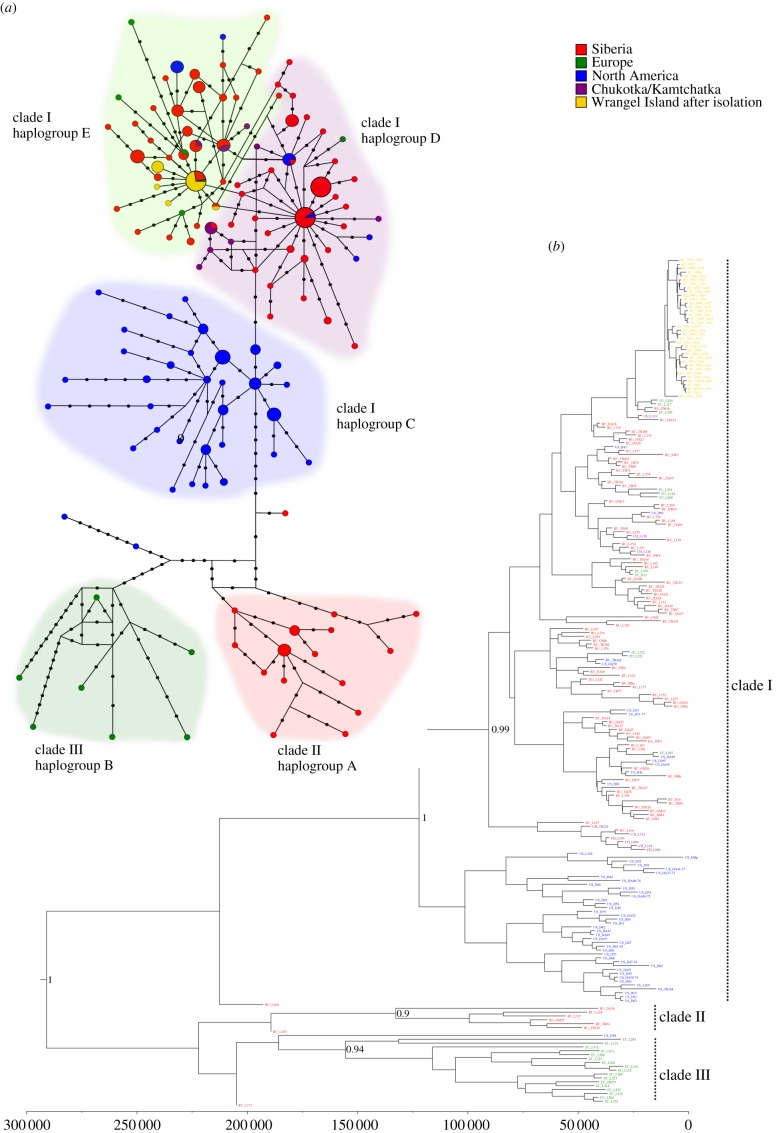
Median-joining haplotype network and Bayesian phylogeny of woolly mammoth mtDNA sequences. (*a*) Haplotype colours indicate their geographical location. Shaded areas correspond to haplogroups as in Debruyne *et al*. [11]. Black dots represent missing haplotypes. Haplotype size is proportional to its frequency within the dataset except for the three most frequent haplotypes within haplogroups D and E that have frequencies above 10. (*b*) The labels at the tips of the phylogeny are coloured according to the geographical origin of the sequences. The timescale on the *x*-axis is in calendar years before present. Bayesian posterior probabilities of major internal nodes above 0.8 are shown.

The complete 741 bp mtDNA sequence was successfully amplified for 56 out of 88 woolly mammoth specimens. Owing to low DNA preservation, the remaining specimens yielded partial sequences. Of these, only 16 could be sequenced for a short 79 bp fragment that contains polymorphic sites informative for clade identification (see electronic supplementary material, table S1). Twenty-nine novel haplotypes were identified (figure 1*a* and electronic supplementary material, figure S3). These sequences together with previously published homologous mtDNA sequences (see electronic supplementary material, table S2) comprised a total dataset of 320 sequences.

### (b) Genetic structure and demographic change

Bayesian phylogenetic analyses using MrBayes and BEAST produced very similar topologies (figure 1*b* and electronic supplementary material, figures S4 and S5). Three major mtDNA clades were identified, each supported by moderate to high posterior probability. Of the three clades, clades I and II have been described earlier [6,10], while we here show that clade III is a lineage containing specimens from Europe. Three specimens from northern Yakutia (northeastern Siberia) that were stratigraphically dated to the LMP were found to be basal to the three clades (figure 1*b* and electronic supplementary material, figures S4 and S5). The date randomization test indicated that the dataset contained sufficient temporal signal for meaningful estimation of molecular rates and dating [19]. Tip-dating in BEAST produced molecular dates for these individuals between *ca* 175 and 200 kyr BP (see electronic supplementary material, table S5).

**Figure 2.**
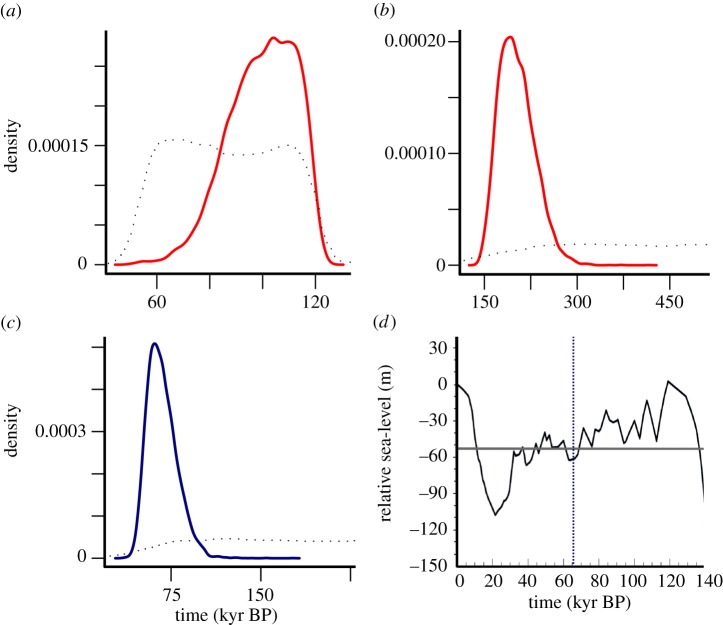
Posterior distributions for the time parameters of models 1B, 2A and relative sea-level (RSL) variation in the Bering Strait. (*a*) Expansion time (T_0_) and (*b*) split time (T_1_) of the three populations in model 1B. (*c*) Split time (T_1_) between the North American and Eurasian population carrying mtDNA clade I in model 2A. Time is given in calendar years before present. The thick red and blue lines show the posterior density curve, and the dotted lines show the prior distribution. (*d*) Figure redrawn from Hu *et al*. [41]. The thick black line represents the RSL and the horizontal grey line shows the current Bering Strait depth. RSL below the grey line indicates exposure of the Bering Strait. The blue vertical dashed line indicates the median of the posterior distribution of the split time (T_1_) between the North American and Eurasian population in model 2A.

Coalescent simulations indicated that the model including a bottleneck followed by exponential growth for the three populations fits the observed data better than the model without growth (BF = 21–70 and electronic supplementary material table S6). Analysis of posterior distributions with the neural networks regression algorithm suggested that the three populations split at *ca* 196 kyr BP (95% credibility interval (CI): 156–261 kyr BP) and went through a contemporaneous demographic expansion at *ca* 121 kyr BP (95% CI: 80–148 kyr BP; figure 2 and electronic supplementary material, table S7). Although the 95% CI of the expansion time is relatively wide, its median is close to the end of the last interglacial period (Marine Isotope Stage (MIS) 5e), suggesting population isolation in interglacial refugia as a likely explanation for the observed divergent mitochondrial lineages. Analysis of additional simulations with a generation time of 20 years (instead of 15 years) produced very similar posterior distributions for split and expansion time (for details, see the electronic supplementary material, table S8).

### (c) Range dynamics of clade I woolly mammoths

Most North American sequences clustered within a distinct subclade of clade I (haplogroup C in figure 1 and electronic supplementary material, figures S3–S5). Earlier studies had suggested a North American origin of this clade [6,11]. According to this hypothesis, the star-like patterns in the main subclade of clade I (haplogroups D and E in figure 1*a*) could suggest that founder effects took place in Eurasia after the dispersal of North American clade I mammoths across Beringia. We estimated the split time between the North American and Eurasian populations carrying mtDNA clade I to *ca* 66 kyr BP (95% CI: 4996 kyr BP; figure 2*c*; electronic supplementary material, table S7), which could correspond to the time when North American mammoths dispersed into Eurasia. It is notable that the two oldest dated clade I specimens found in eastern Siberia, with radiocarbon estimates of greater than 60 kyr BP (hap 2 and hap 5 in electronic supplementary material, figure S3 and see tables S1 and S2 for details), carry haplogroups identical to, and two mutational steps from, the modal haplotype in haplogroup D, which is what would be expected if this modal haplotype represents the founding lineage of the expansion that took place after the dispersal event.

The presence of North American sequences outside haplogroup C, in the main subclade of clade I (figure 1) could be indicative of post-colonization gene flow in an eastwards direction [11]. The simulations, however, provided higher support (BF > 200; electronic supplementary material, table S6) for the model without gene flow from the Eurasian population to the North American population after their split (model 2A in electronic supplementary material, figure S2). Thus, an alternative explanation could be that these lineages represented polymorphisms that were already present in the ancestral population in North America.

### (d) Genetic turnover events

**Figure 3.**
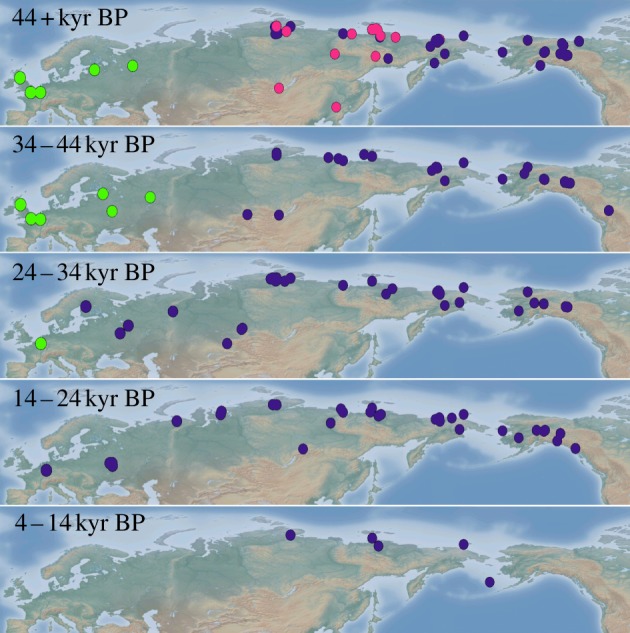
Spatial distribution of radiocarbon-dated and genetically analysed mammoth specimens. Dates are given in calendar years before present. Colours indicate clade membership of the specimens: clade I; purple, clade II; pink, clade III; green.

Following the expansion from North America to Eurasia, clade I appears to have been sympatric with clade II in Central and East Siberia until the demise of the latter (figure 3; see also [6,10]). Based on finite radiocarbon dates from clade II specimens, it seems that this clade disappeared around 45 kyr BP (although it should be noted that one clade II specimen has yielded an infinite date of more than 33 000 ^14^C years, roughly corresponding to more than 37.5 kyr BP). Furthermore, clade I woolly mammoths continued expanding to the west, all the way to Europe, where they replaced the endemic population carrying mtDNA clade III (figure 3). Clade III disappeared from the fossil record at *ca* 34 kyr BP, whereas clade I seems to have made its first appearance in Europe at *ca* 32 kyr BP (see electronic supplementary material, table S1).

### (e) Bayesian analysis of changes in population size through time

**Figure 4.**
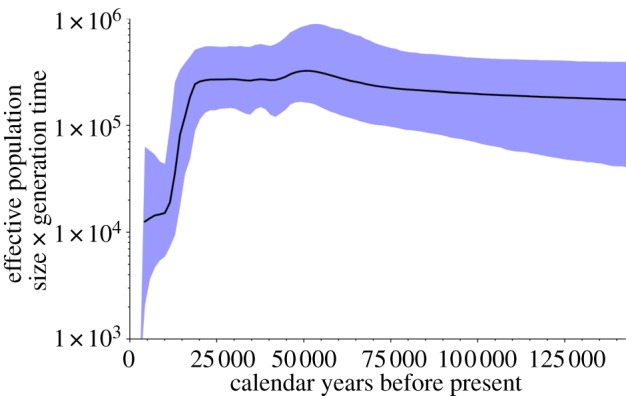
BSP of all dated woolly mammoth specimens. Effective population size (*N*_ef_) on the *y*-axis is biased by a factor of generation time assumed to 15 years. The thick solid line shows the average product of effective population size and generation time and the shaded area represents the 95% HPD. (Online version in colour.)

The Bayesian skyline plot (BSP) showed a severe and sudden decline in *N*_ef_ during MIS 2, starting at *ca* 20–15 kyr BP and ending early in MIS 1, at *ca* 10 kyr BP (figure 4). The observed reduction in *N*_ef_ was at least 10-fold from *ca* 20 000 individuals (95% highest posterior density (HPD): 40 000–10 000) to *ca* 1000 individuals (95% HPD: 3000–400) assuming an average generation time of 15 years. Even though the HPD intervals are wide for the periods preceding and following the bottleneck, *N*_ef_ seems to have remained relatively constant until this decline.

## 4. Discussion

The last interglacial, known in Europe as the Eemian and equated with MIS 5e (130–116 kyr BP), was a period characterized by climate at least as warm as today [42]. Tundra habitats in mainland Europe and Siberia were partly replaced by mixed or more open forests and sea levels were as high as, or even higher than that at present [42,43]. Species adapted to the cold, dry and open conditions of the steppe–tundra, such as the woolly mammoth, were likely affected by these environmental changes through range contractions and demographic declines [44]. Previous research employing back-casted climate models has proposed that habitat suitable for the woolly mammoth in Eurasia was severely reduced during the Eemian interglacial, restricting the mammoth's geographical range to a few northern areas [45]. Our genetic results appear to be consistent with this hypothesis, as they indicate that demographic expansions, presumably preceded by bottlenecks, took place around the end of the last interglacial (*ca* 121 kyr BP; figure 2*a*). This suggests that woolly mammoth populations were constrained in refugia during the Eemian interglacial, and subsequently expanded in population size as well as geographically across Eurasia and North America as the climate became cooler and drier. This implies that severe bottlenecks at the onset of interglacials may have been a recurrent feature in the woolly mammoth's evolutionary history, and consequently lends support for the hypothesis that the end-Pleistocene retraction of the woolly mammoth from most of its range was driven by climate change.

From a genetic perspective, isolation in interglacial refugia and the subsequent demographic expansions indicated in our analyses could also provide an explanation for the existence of three distinct mtDNA clades during the Late Pleistocene. Divergent mtDNA lineages have been detected in many other taxa, and it has been argued that these most likely evolved owing to long-term allopatric isolation in glacial refugia [46,47]. As a cold-adapted species, the opposite pattern is expected for the woolly mammoth [48]. The presence of three distinct and highly diverged woolly mammoth mtDNA lineages (figure 1*b*) suggests that at least three separate interglacial refugia may have existed during the Eemian. The European distribution of clade III mammoths points to the existence of an interglacial refugium in western Eurasia, whereas the restricted distribution of clade II mammoths (figure 3) and American origin of clade I [6,11] imply two additional refugia, in northern Siberia and North America, respectively. However, the observed phylogenetic pattern should be interpreted with caution because it is based on mtDNA, which is a single non-recombining locus [49].

Our estimate of split time for the three populations, representing the three mtDNA clades, (*ca* 196 kyr BP) appears to be much younger than the tip calibrated divergence time estimated by Gilbert *et al.* [10] (*ca* 1 Ma). However, coalescence times of gene lineages (e.g*.* estimated in BEAST) typically predate population split times [50]. Moreover, it should be noted that tip calibration of the molecular clock requires a sufficient temporal signal in the data [19,51]. In contrast to our dataset, it has been shown that the full mtDNA genomes presented in Gilbert *et al*. [10] lacked such signal [19], likely owing to small sample size and limited temporal coverage. Thus, more complete mtDNA genomes need to be sequenced in order to further resolve the divergence times among the mtDNA clades.

Three specimens in our dataset from Yakutia (eastern Siberia) were stratigraphically dated to the LMP. These were phylogenetically basal to, and thus fall outside the diversity of, the three mtDNA clades (figure 1*b* and electronic supplementary material, figures S4 and S5). The ages of these specimens, as estimated in BEAST, were approximately 175–200 kyr BP, and thus fall within the LMP (see electronic supplementary material, table S5). These specimens therefore predate the time to the most recent common ancestor of each mtDNA clade (figure 1*b*) as well as the expansion time of the three populations belonging to each clade, inferred from the simulations (approx. 121 kyr BP; figure 2*a*), and could therefore represent ancestral variation that existed before the Eemian population bottlenecks.

The decrease in temperatures and retreat of forests that occurred during the early stages of the last glaciation likely allowed for fragmented mammoth populations to expand their range [45]. Following the refugial scenario discussed above, woolly mammoths carrying mtDNA clade I would have expanded across northern North America, whereas clade II mammoths spread into Central and East Siberia and clade III mammoths colonized most of Europe. These regions were thus initially inhabited by discrete woolly mammoth populations, at least with respect to maternal gene flow, for thousands of years.

The onset of the last glaciation led to a gradual accumulation of ice in the Scandinavian and North American ice sheets, which in turn induced a decrease in sea levels [52]. The Bering Land Bridge that had been inundated owing to elevated sea levels during the Eemian became exposed, providing a corridor that connected northeast Siberia and Alaska. Previous studies have suggested that woolly mammoths belonging to clade I originated in North America and colonized Eurasia by crossing over the Bering Land Bridge but the timing of this expansion has been debated [6,11]. Using coalescent simulations, we estimated that the population split between North American and Eurasian mammoths carrying mtDNA clade I occurred at *ca* 66 kyr BP (figure 2*c*), a timing that could represent the westward dispersal of North American woolly mammoths into Eurasia. This coincides with the first exposure of the Bering Land Bridge since the penultimate glacial period [41] (figure 2*d*). Therefore, climate-induced sea-level changes could have promoted the expansion of clade I mammoths into Eurasia. It should be noted that our simulations did not explicitly test the hypothesis that woolly mammoths carrying mtDNA clade I from North America colonized Eurasia [6,11] but under this assumption inferred the timing of the presumable dispersal event.

Gene flow in the opposite direction, from Eurasia to North America after the populations split, was not supported by the simulations (see electronic supplementary material, table S6). This implies that the existence of sequences belonging to haplogroups D and E in North America [11] is owing to incomplete lineage sorting in the North American population. Assuming that founder effects took place in Siberia during the initial colonization, higher genetic drift would be expected in the Eurasian population compared with the North American population. This could be the reason why we observed an asymmetry in lineage sorting among the two populations.

If North American woolly mammoths expanded into Siberia, they would have encountered and likely interbred with genetically distinct individuals (carrying mtDNA clade II) that resided in Central and East Siberia. The two mtDNA clades subsequently coexisted in sympatry for more than 20 kyr until clade II disappeared at around 40 kyr BP (figure 3), either owing to selection or, more likely, genetic drift [6,10]. Furthermore, our data show that the geographical distribution of clade I continued to expand westwards, reaching European Russia at *ca* 32 kyr BP (figure 3). This first appearance of mammoths carrying mtDNA clade I in Europe appears to coincide with the disappearance of the endemic population in Europe (carrying mtDNA clade III) at *ca* 34 kyr BP (figure 3). However, in contrast to the genetic turnover in northeast Siberia, there is no evidence for a temporal overlap between clades I and III in Europe, which could indicate that the European population became extinct, and that Europe subsequently was recolonized by Siberian mammoths carrying clade I. There is size variation among mammoths in the last glaciation of Europe, but it does not map clearly to clade membership and may be owing to environmental effects, for example, vegetational productivity [53–55]. In Western Europe, large body size was maintained up to the last populations in the late-glacial [56].

It is interesting to note that similar population turnovers have been identified in cave bears from Central Europe at *ca* 31 kyr BP [2] and collared lemmings from northwestern Europe between *ca* 43–32 kyr BP [5]. The possible synchrony of these events could indicate that the turnovers were driven by regional changes in the environment. These population turnovers also appear to coincide approximately with the earliest findings of Gravettian culture in northwest Europe, around 34 kyr BP [57,58]. In the case of the mammoth, the extinction of clade III might even be associated with the emergence of the Gravettian culture itself, although this seems less likely as this would not explain how Siberian mammoths could have immediately recolonized Europe, and then survived there for at least another 15 kyr.

Spatio-temporal changes in fossil abundance suggest that northern populations of woolly mammoth generally declined between about 27 and 18 kyr BP (broadly the LGM), whereas populations in Central and South Siberia initially increased [16,59], and subsequently declined during the Bølling–Allerød interstadial. The Younger Dryas (*ca* 12.9–11.7 kyr BP) was associated with extirpation in North America and southern Siberia, but temporary expansion in northern Siberia and into northeast Europe where mainland populations survived into the early Holocene (until *ca* 11 kyr BP) [9,16]. Such complex range contractions and asynchronous local extinctions resulted in the final demise of the mainland populations [9,60]. Previous studies have attempted to recover a signal of population decrease or the final extinction using genetic data but failed to do so ([6,11,59] but see [13]). Lorenzen *et al.* [36] simulated and compared different demographic models and found support for a population increase before the LGM (*ca* 26 kyr BP) in Eurasian mammoths. However, no significant changes in population size were observed when looking at temporal dynamics of global *N*_ef_, or could be inferred from modelling the potential inhabitable range size through time. In contrast to these previous studies, we identified a marked end-Pleistocene population reduction (figure 4). Larger sample size with thorough temporal coverage, together with the inclusion of mammoth sequences from the post-bottleneck population on Wrangel Island in our dataset, offered the resolution required to capture significant changes in *N*_ef_ [59]. We suggest that the revealed drastic drop in female effective population size most probably reflects the cumulative effects of the Late Pleistocene decline in most of the geographical range of woolly mammoths as well as the population bottleneck on Wrangel Island [13].

The results from this study reveal that the Late Pleistocene history of the woolly mammoth was characterized by a complex series of demographic changes, range expansions and clade replacements. Thus, while the high prevalence of mammoths in the fossil record might imply a stable and abundant species, populations of the woolly mammoth appear to have been highly dynamic. Both genetic data and the radiocarbon record indicate a dramatic final demographic decline at the end of the last glaciation. However, our results suggest that this decline was mirrored by a similar decline during the previous interglacial, a pattern that has also previously been observed in other cold-adapted taxa, such as reindeer [61], arctic fox [62] and polar bear [63]. It thus seems likely that environmental changes played a significant role in shaping the woolly mammoth's demographic history, with warm periods restricting the amount of available habitat and cold periods leading to population expansions, both owing to increases in the amount of steppe–tundra and through sea-level-driven exposure of the Bering Land Bridge. Resolving why the woolly mammoth survived in refugia during earlier interglacials, but not during the Holocene, may thus provide the key to understand the mechanism behind its final extinction.
